# Serum calcium-magnesium-phosphate-potassium metabolic dysregulation in nephrolithiasis: predictive model development and mechanistic insights

**DOI:** 10.3389/fphys.2026.1835199

**Published:** 2026-08-19

**Authors:** Yunhan Wang, Caitao Dong, Wenbiao Liao, Sixing Yang

**Affiliations:** 1 Department of Urology, Renmin Hospital of Wuhan University, Wuhan, Hubei, China; 2 Hubei Provincial Clinical Research Center for Urolithiasis, Wuhan, Hubei, China

**Keywords:** calcium magnesium ratio, calcium phosphate product, hypokalemia, multivariable predictive model, nephrolithiasis

## Abstract

**Purpose:**

To explore the pathophysiological interplay of serum calcium, magnesium, phosphate, and potassium (Ca, Mg, P, K) homeostasis in nephrolithiasis and establish an integrated risk prediction framework incorporating these routine homeostatic biomarkers.

**Methods:**

This retrospective, hospital-based case-control study enrolled 611 patients with radiologically confirmed nephrolithiasis and 624 strict gender-matched controls to eliminate sex-related baseline biases. Fasting morning serum biochemical data were analyzed. Multivariable logistic regression identified core risk determinants. Model performance was rigorously validated through receiver operating characteristic (ROC) analysis, calibration curves, and decision curve analysis (DCA), followed by the construction of a diagnostic nomogram.

**Results:**

The predictive model based on electrolyte indices demonstrated exceptional discriminative capacity (AUC = 0.812, 95% CI: 0.789–0.836) and high calibration accuracy (Brier score = 0.177, Hosmer-Lemeshow p = 0.852). Key independent predictors included an elevated serum calcium magnesium ratio (Ca/Mg, aOR = 5.50), increased calcium phosphate product (Ca×IP, aOR = 1.82), hypokalemia (aOR = 0.13), BMI ≥ 24, and reduced hemoglobin levels. DCA confirmed the model’s net clinical benefit across a wide range of threshold probabilities.

**Conclusion:**

Dysregulation of serum Ca, Mg, P, K homeostasis is strongly associated with nephrolithiasis. Our nomogram may offer a complementary, blood-based initial assessment when 24-hour urine testing is not feasible, pending further validation.

## Introduction

Nephrolithiasis represents a global health challenge, characterized by a 5-year recurrence rate surpassing 50% and imposing substantial clinical and economic burdens (Abufaraj et al., 2021; Tan et al., 2024). Calcium-based calculi dominate the epidemiological landscape, comprising 75%–80% of all stone types. Of these, calcium oxalate (CaOx) stones constitute the majority (>60%), while calcium phosphate (CaP) variants account for 15%–20% (Coe et al., 2005; Ferraro et al., 2015). Despite diagnostic advances in stone composition analysis and 24-hour urinary metabolic profiling, 30%–40% of cases exhibit no identifiable metabolic derangements, creating critical barriers to personalized prevention protocols (Eisner et al., 2012; Ferraro et al., 2024).

Traditional research has predominantly focused on isolated risk factors (e.g., hypercalciuria, hypocitraturia), while neglecting the synergistic effects of dynamic electrolyte interactions involving calcium (Ca), magnesium (Mg), phosphate (P), and potassium (K) in the pathogenesis of nephrolithiasis (Coe et al., 2016; Moe and Xu, 2018; Peerapen and Thongboonkerd, 2023). These ions exhibit intricate regulatory crosstalk: magnesium competitively inhibits calcium oxalate crystallization (Bushinsky et al., 2000), and high phosphate intake reduces intestinal calcium phosphate binding, thereby enhancing calcium absorption and urinary calcium excretion. Low-phosphate diet combined with potassium citrate therapy effectively decreases urinary phosphate excretion and inhibits stone formation (Huang et al., 2023). Potassium imbalance indirectly disrupts calcium magnesium transport through modulation of Na^+^/K^+^-ATPase activity (Pierre and Xie, 2006; Hodeify et al., 2021). Critically, current risk stratification frameworks fail to systematically incorporate these multidimensional interactions, suggesting that conventional calcium-centric models inadequately capture the pathophysiological complexity of stone formation. Composite indices, such as the calcium magnesium ratio (Ca/Mg) and the calcium phosphate product (Ca×IP), can integrate synergistic or antagonistic ion interactions, potentially revealing subtle homeostatic disruptions that are undetectable when examining any single electrolyte in isolation. These ratios may therefore serve as more sensitive proxies for the overall functional state of the electrolyte homeostasis.

This hospital-based case-control study aims to evaluate the predictive power of composite indices, specifically the calcium magnesium ratio and the calcium phosphate product, in comparison to traditional single-ion measurements. Our objective is to determine if dysregulation of these four interrelated electrolytes is associated with stone formation, thereby establishing a risk prediction model based on multi-element equilibrium. The outcomes of this research are expected to lay a foundation for tailored nutritional intervention strategies. Moreover, given that 24-hour urine collection exhibits extremely poor compliance in emergency or initial consultation settings, there is an urgent clinical need for a rapid, reliable early-warning tool based on routine venous blood sampling. This study aims to bridge this critical gap. Although individual electrolyte ratios have been studied previously, the combined use of Ca/Mg and Ca×IP together with serum potassium in a single predictive model for nephrolithiasis has not been reported.

## Methods

### Study design and data sources

We conducted a hospital-based case-control study at Renmin Hospital of Wuhan University from 2023 to 2025, incorporating participants from both case and control groups. Sociodemographic and fasting morning serum biochemical data were retrospectively acquired from the hospital’s electronic medical records (EMR). Ethical approval for this retrospective study was granted by the Ethics Committee of Renmin Hospital of Wuhan University (approval number: WDRY2023-K183), which also waived the requirement for written informed consent.

### Participant selection

Inclusion Criteria for Cases: (1) Age >18 years; (2) Radiologically confirmed nephrolithiasis (stone diameter ≥2 mm) by urinary tract ultrasonography or non-contrast computed tomography (NCCT). The stone-free status of the control group was also rigorously confirmed using the same imaging modalities to avoid misclassification bias.

Exclusion Criteria: History of primary hyperoxaluria, hyperparathyroidism, gastrointestinal disorders/surgery, autoimmune diseases, chronic kidney disease, urinary tract abnormalities/obstruction, malignancies, or other conditions potentially interfering with calcium phosphate metabolism.

### Sample size

**Figure 1:**
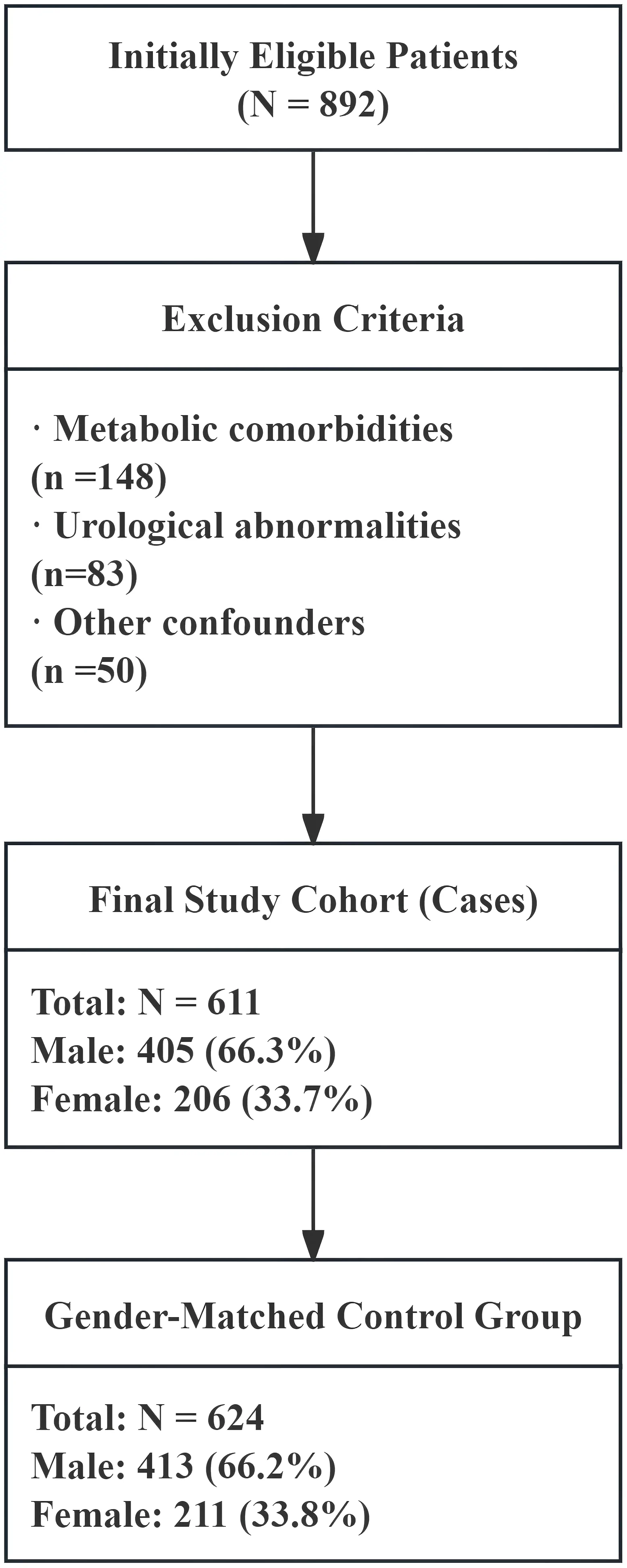
Flowchart of case and control selection process with gender matching.

As illustrated in **Figure 1**, out of the 892 initially eligible nephrolithiasis patients, 281 were excluded due to various factors: metabolic comorbidities (n=148), urological abnormalities (n=83), and other confounders (n=50), yielding 611 patients (405 males, 206 females). To completely eliminate baseline discrepancies in serum creatinine levels and other metabolic parameters driven by sex, we adopted a strict gender-matched strategy when recruiting the control group (624 subjects: 413 males, 211 females). The slight difference in sample size between groups (611 vs. 624) is due to the rigorous exclusion criteria applied to cases; the numbers remain closely balanced.

The required sample size was determined *a priori* based on the rule of thumb for logistic regression of at least 10 events per predictor variable (EPV). Assuming a nephrolithiasis prevalence of approximately 10% in the source population and anticipating a model with up to 15 candidate predictors, a minimum of 150 cases was required. Our final model includes 7 independent predictors, for which 611 cases provide ample statistical power (EPV > 80). The final sample size of 611 cases and 624 controls significantly exceeds this minimum, ensuring stable estimates and robust model development.

### Statistical analysis

Data processing was conducted using Excel 2021 and SPSS 26.0. The normality of continuous variables was assessed using the Shapiro-Wilk test. Normally distributed data are presented as mean ± SD, while non-normally distributed data are expressed as median (IQR). Intergroup comparisons were performed using independent t-tests for normally distributed data and the Kolmogorov-Smirnov test for non-normal data. Categorical variables are reported as frequency (%) with differences assessed using χ² tests. Binary logistic regression modeling proceeded in two phases: univariate screening of significant variables (P < 0.05), followed by forward stepwise multivariable adjustment to calculate adjusted odds ratios (aORs) with 95% confidence intervals (CIs).

Statistical analyses were conducted utilizing R software (4.4.2) and its integrated development environment, incorporating specialized packages for predictive modeling and visualization. The analytical workflow comprised three sequential phases: model construction, performance validation, and clinical application.

For nomogram development, multivariable regression coefficients were proportionally scaled to establish variable-specific scoring thresholds. Predictor variables were vertically aligned to facilitate point summation. The aggregated score was then mapped to probability estimates through coordinate projection onto the outcome risk axis. Model discrimination was assessed using receiver operating characteristic (ROC) analysis, yielding conventional diagnostic performance metrics, including sensitivity, specificity, and area under the curve (AUC). Predictive calibration was rigorously evaluated through comparisons of observed versus predicted probabilities, employing bootstrap-corrected calibration curves. Clinical utility was assessed using threshold-dependent net benefit quantification across clinically relevant probability ranges through decision curve analysis. Subgroup analyses examined risk heterogeneity based on sex and renal function. All serum biochemical parameters included in the final multivariable model (Ca, Mg, P, K, Cr, Hb, Cl, and derived indices) had no missing data. For other exploratory variables (e.g., ALT, AST, ALB), missingness was less than 3%, and complete-case analysis was applied. All statistical tests were two-tailed, with significance defined at P < 0.05.

## Results

This study involved 611 patients with nephrolithiasis and 624 matched healthy controls. As presented in **Table 1**, a comparison of baseline characteristics indicated no statistically significant differences in gender distribution (males: 66.3% in the stone group versus 66.2% in the control group) or median age (both groups: 55 years) (all P > 0.05). Body mass index (BMI) stratification demonstrated a significantly higher proportion of overweight/obesity in the stone group (32.6% vs. 22.8%, χ² = 18.632, P < 0.001), particularly notable in overweight individuals (29.0% vs. 20.4%). Both groups exhibited comparable smoking rates, alcohol consumption, and prevalence of hypertension/diabetes (all P > 0.05).

**Table 1:** Comparison of demographic and serum biochemical characteristics between nephrolithiasis patients and gender-matched controls.

Characteristics	Case (n=611)	Control (n=624)	t/χ^2^/Z	P^*^
Gender, n (%)			0.001	0.971
Male	405 (66.3)	413 (66.2)		
Female	206 (33.7)	211 (33.8)		
Age, year, Median (IQR)	55 (19)	55 (20)	0.787	0.565
BMI ^a^, n (%)			18.632	< 0.001
Underweight	18 (2.9)	36 (5.8)		
Normal	394 (64.5)	446 (71.5)		
Overweight	177 (29.0)	127 (20.4)		
Obesity	22 (3.6)	15 (2.4)		
Smoke, n (%)	162 (26.5)	145 (23.2)	1.775	0.183
Drink, n (%)	103 (16.9)	117 (18.8)	0.755	0.385
Hypertension, n (%)	181 (29.6)	176 (28.2)	0.302	0.582
Diabetes, n (%)	71 (11.6)	64 (10.3)	0.590	0.442
Ca, mmol/L	2.23 (0.10)	2.23 (0.10)	0.410	0.996
Mg, mmol/L	0.81 (0.09)	0.85 (0.09)	3.414	< 0.001
Ca/Mg ^a^	2.74 (0.31)	2.63 (0.28)	3.693	< 0.001
ALT, U/L	18.00 (14.00)	20.16 (15.00)	1.900	0.001
AST, U/L	20.00 (7.00)	21.21 (7.38)	3.025	< 0.001
ALT/AST	0.94 (0.52)	0.94 (0.46)	0.766	0.600
ALB, g/L	41.90 (5.00)	43.50 (4.60)	3.596	< 0.001
Cr, μmol/L	79.00 (31.00)	66.00 (22.00)	5.401	< 0.001
UA, μmol/L	358.00 (148.00)	337.25 (124.49)	2.042	< 0.001
GLU, mmol/L	4.91 (1.07)	4.91 (0.96)	0.675	0.753
K, mmol/L	3.93 (0.43)	4.10 (0.37)	4.382	< 0.001
Na, mmol/L	140.60 (2.70)	140.20 (2.70)	1.528	0.019
Cl, mmol/L	106.80 (3.10)	106.10 (3.20)	2.178	< 0.001
IP, mmol/L	1.14 (0.25)	1.11 (0.24)	1.219	0.103
Ca×IP ^a^, mmol^2^/L^2^, Mean ± SD	2.42 ± 0.47	2.26 ± 0.44	5.972	< 0.001
eGFR ^a^, mL/min	91.64 (31.15)	101.58 (20.20)	5.189	< 0.001
Hb, g/L	137.14 ± 22.00	140.97 ± 16.91	-4.082	< 0.001
HCT, L/L	0.41 ± 0.04	0.42 ± 0.05	-4.722	< 0.001

^a^ BMI, body mass index; Ca/Mg, serum calcium magnesium ratio; Ca×IP, serum calcium phosphate product; eGFR, estimated glomerular filtration rate; IP, inorganic phosphate.

^*^ P < 0.05 based on Student’s t-test, Mann-Whitney U test, or Kolmogorov-Smirnov test as appropriate.

Serum biochemical analysis identified significantly lower Mg levels in the stone group (0.81 vs. 0.85 mmol/L, P < 0.001) with elevated calcium magnesium ratio (Ca/Mg: 2.74 vs. 2.63, P < 0.001). Parameters indicative of hepatic function showed reduced ALT (18.00 vs. 20.16 U/L) and AST (20.00 vs. 21.21 U/L) in stone formers (both P < 0.01), though ALT/AST ratio remained comparable. Notably, serum albumin (ALB) was significantly decreased in the stone group (41.90 vs. 43.50 g/L, P < 0.001), while renal function markers demonstrated significant abnormalities: elevated creatinine (Cr: 79.00 vs. 66.00 μmol/L), increased uric acid (UA: 358.00 vs. 337.25 μmol/L), and reduced estimated glomerular filtration rate (eGFR: 91.64 vs. 101.58 mL/min/1.73 m²) (all P < 0.001).

Electrolyte profiling revealed significantly lower potassium (K: 3.93 vs. 4.10 mmol/L) alongside elevated sodium (140.60 vs. 140.20 mmol/L) and chloride (106.80 vs. 106.10 mmol/L) in the stone group (all P < 0.05). The calcium phosphate product (Ca×IP: 2.42 vs. 2.26 mmol²/L², P < 0.001) was markedly increased in the stone group. Hematological parameters showed decreased hemoglobin (Hb: 137.14 vs. 140.97 g/L) and hematocrit (HCT: 0.41 vs. 0.42) in the stone group (both P < 0.001). No significant intergroup differences were observed in total serum calcium (Ca), fasting glucose (GLU), or inorganic phosphate (IP) levels (all P > 0.05).

Both univariate and multivariable logistic regression analyses revealed independent associations between overweight status, elevated calcium magnesium ratio (Ca/Mg), and nephrolithiasis risk (**Table 2**). Univariate analysis identified 13 significant predictors including overweight (OR = 1.578), reduced Mg (OR = 0.001), elevated Ca/Mg (OR = 7.983), elevated ALB (OR = 0.871), elevated Cr (OR = 1.037), elevated UA (OR = 1.002), reduced K (OR = 0.166), elevated Cl (OR = 1.103), elevated calcium phosphate product (Ca×IP, OR = 2.128), and reduced Hb (OR = 0.986), all demonstrating statistical significance (P < 0.05).

**Table 2:** Univariate and multivariable logistic regression analyses of serum homeostatic indicators associated with nephrolithiasis risk.

Variable	Univariate			Multivariable		
	Crude OR^a^	95% CI ^a^	P-value^*^	Adjusted OR	95% CI	P-value^*^
Gender, n (%)						
Male	1.000	1.000	-			
Female	0.996	0.786–1.260	0.971			
Age, year, Median (IQR)	1.002	0.994–1.011	0.564			
BMI ^a^, n (%)						
Underweight	0.566	0.346–1.013	0.055	0.630	0.312–1.272	0.197
Normal	1.000	1.000	-	1.000	1.000	-
Overweight	1.578	1.210–2.057	< 0.001	1.585	1.160–2.166	0.004
Obesity	1.660	0.849–3.245	0.138	1.757	0.803–3.841	0.158
Smoke, n (%)						
No	1	1	-			
Yes	1.192	0.920–1.543	0.183			
Drink, n (%)						
No	1	1	-			
Yes	0.879	0.656–1.177	0.385			
Hypertension, n (%)						
No	1	1	-			
Yes	1.071	0.838–1.370	0.583			
Diabetes, n (%)						
No	1	1	-			
Yes	1.150	0.804–1.646	0.443			
Ca, mmol/L	2.453	0.647–9.304	0.187			
Mg, mmol/L	0.001	0.001–0.003	< 0.001			
Ca/Mg ^a^	7.983	4.851–13.136	< 0.001	5.501	3.105–9.745	< 0.001
ALT, U/L	0.997	0.993–1.002	0.298			
AST, U/L	0.995	0.986–1.003	0.202			
ALT/AST	0.880	0.666–1.162	0.366			
ALB, g/L	0.871	0.842–0.901	< 0.001			
Cr, μmol/L	1.037	1.030–1.043	< 0.001	1.048	1.040–1.057	< 0.001
UA, μmol/L	1.002	1.001–1.004	< 0.001			
GLU, mmol/L	1.045	0.965–1.131	0.279			
K, mmol/L	0.166	0.114–0.242	< 0.001	0.128	0.083–0.197	< 0.001
Na, mmol/L	1.059	1.006–1.115	0.028			
Cl, mmol/L	1.103	1.055–1.154	< 0.001	1.064	1.007–1.124	0.027
IP, mmol/L	1.528	0.840–2.779	0.165			
Ca×IP^a^, mmol^2^/L^2^, Mean ± SD	2.128	1.649–2.746	< 0.001	1.822	1.347–2.466	< 0.001
eGFR ^a^, mL/min	0.964	0.957–0.970	< 0.001			
Hb, g/L	0.986	0.979–0.993	< 0.001	0.985	0.976–0.994	< 0.001
HCT, L/L	0.003	0.001–0.032	< 0.001			

^a^ BMI, body mass index; Ca/Mg, serum calcium magnesium ratio; Ca×IP, serum calcium phosphate product; eGFR, estimated glomerular filtration rate; OR, odds ratio; CI, confidence interval.

^*^ P < 0.05 denotes statistical significance in the logistic regression models.

Multivariable logistic regression analysis was performed using forward stepwise selection, incorporating variables that demonstrated significant associations (P < 0.05) in preliminary univariate screening. Following adjustments for these significant predictors, seven variables remained independently associated with the outcome: overweight (aOR = 1.585, 95% CI: 1.160–2.166), elevated Ca/Mg (aOR = 5.501, 95% CI: 3.105–9.745), elevated Cr (aOR = 1.048, 95% CI: 1.040–1.057), reduced K (aOR = 0.128, 95% CI: 0.083–0.197), elevated Cl (aOR = 1.064, 95% CI: 1.007–1.124), elevated Ca×IP (aOR = 1.822, 95% CI: 1.347–2.466), and reduced Hb (aOR = 0.985, 95% CI: 0.976–0.994), with all P < 0.05. Notably, four parameters demonstrated particularly strong clinical associations: Ca/Mg elevation, K reduction, Ca×IP elevation, and overweight status exhibited particularly strong associations with the outcome. No significant associations were observed for obesity (aOR = 1.757, P = 0.158) or underweight status (aOR = 0.630, P = 0.197).

**Figure 2:**
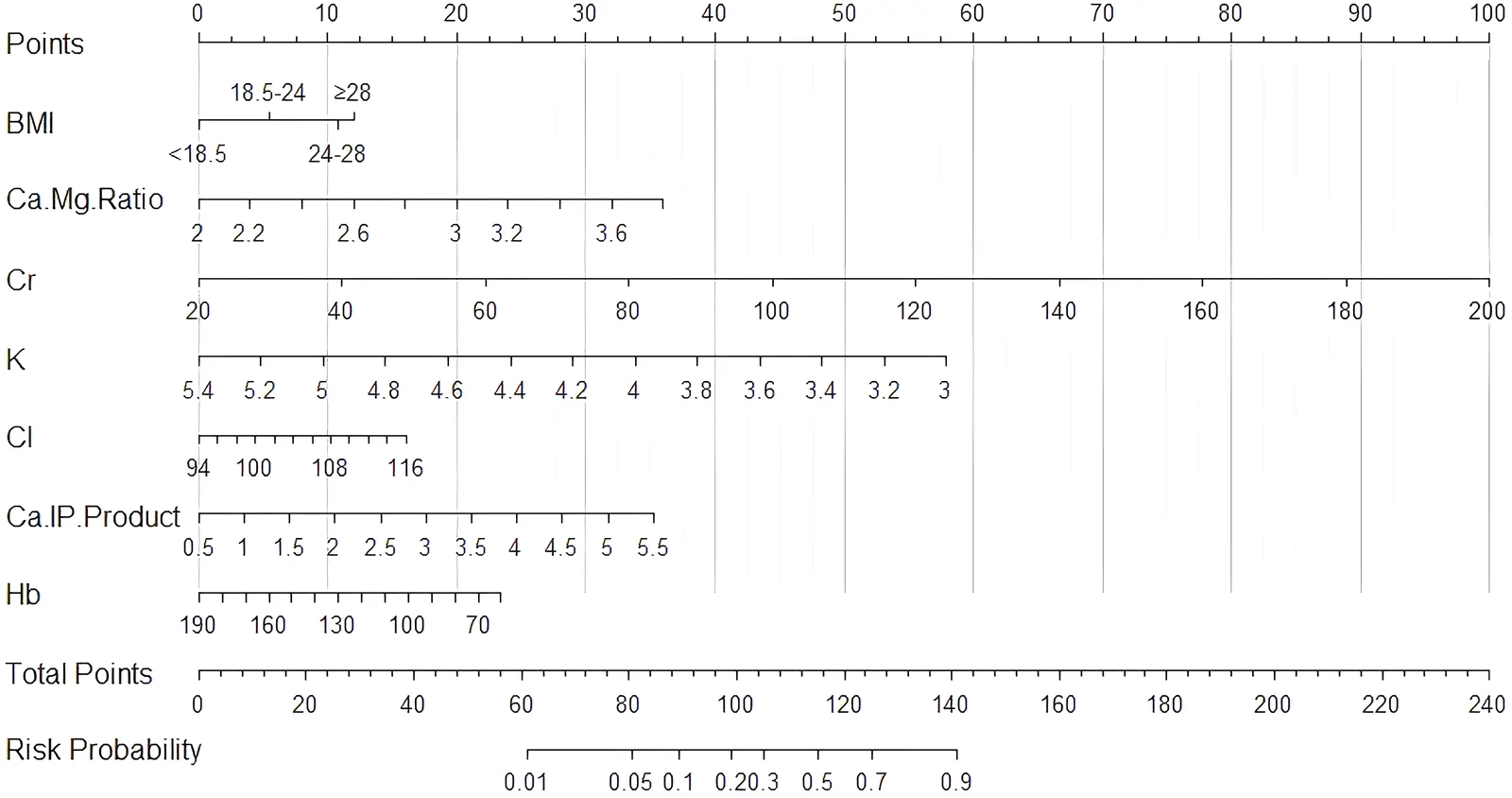
The risk prediction nomogram developed in this study integrates clinical parameters derived from a multivariable logistic regression model, including BMI, Ca/Mg ratio, serum creatinine (Cr), blood potassium (K), blood chloride (Cl), calcium phosphate product (Ca×IP), and hemoglobin (Hb). Variable-specific scores are standardized based on regression coefficients, with the total score mapped to a risk probability axis (0.01–0.9). The model exhibits robust discrimination (C-index = 0.812).

This study developed a clinical prediction nomogram (**Figure 2**) for visual risk assessment, demonstrating strong discriminative power (C-index = 0.812) and good calibration (Brier score = 0.177). Seven key predictors were integrated into the model: calcium-magnesium ratio imbalance, overweight status (BMI ≥24 kg/m^2^), elevated serum creatinine, increased calcium-phosphate product, hypokalemia, elevated serum chloride, and decreased hemoglobin levels. The nomogram enables rapid estimation of individualized risk probabilities by summing scores assigned to each variable. A total score of 240 corresponds to a high-risk probability, providing a practical tool for early identification of high-risk patients and formulation of clinical intervention strategies.

**Figure 3:**
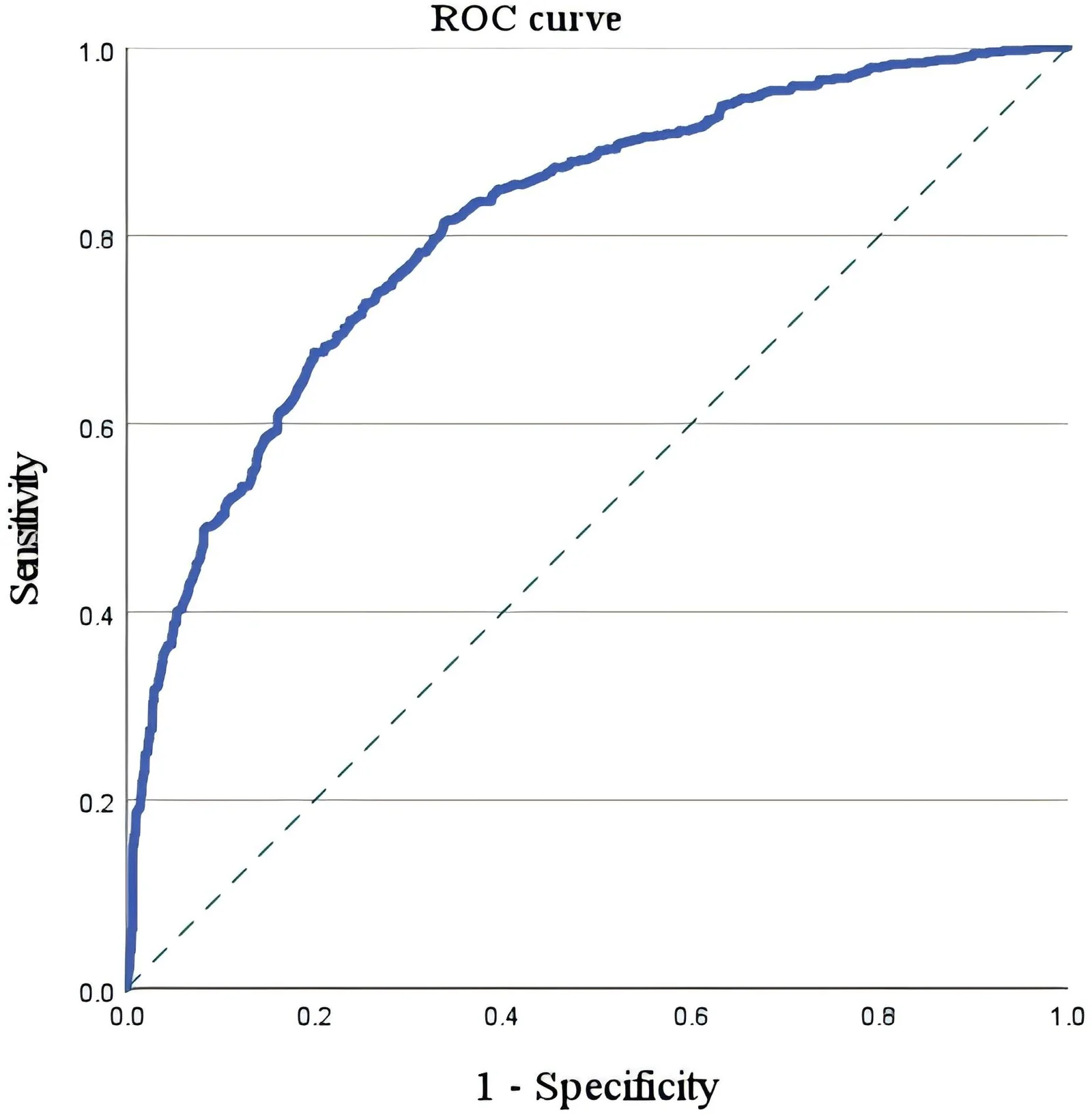
Receiver operating characteristic (ROC) curve derived from the multivariable logistic regression model for predicting nephrolithiasis risk. The area under the curve (AUC) was 0.812 (95% CI: 0.789-0.836, P < 0.001), indicating a substantial discriminative ability to differentiate between cases and controls. The dotted diagonal line represents the reference line for random chance (AUC = 0.5).

As shown in **Figure 3**, the multivariable model demonstrated robust discriminative capacity with an AUC of 0.812 (95% CI: 0.789–0.836), significantly superior to random classification (P < 0.001). At the Youden index-optimized cutoff, sensitivity and specificity reached 78.9% and 73.6%, respectively. The AUC standard error (0.012) and exclusion of the null value (0.5) from the CI confirmed model robustness, supporting its clinical applicability for risk assessment.

**Figure 4:**
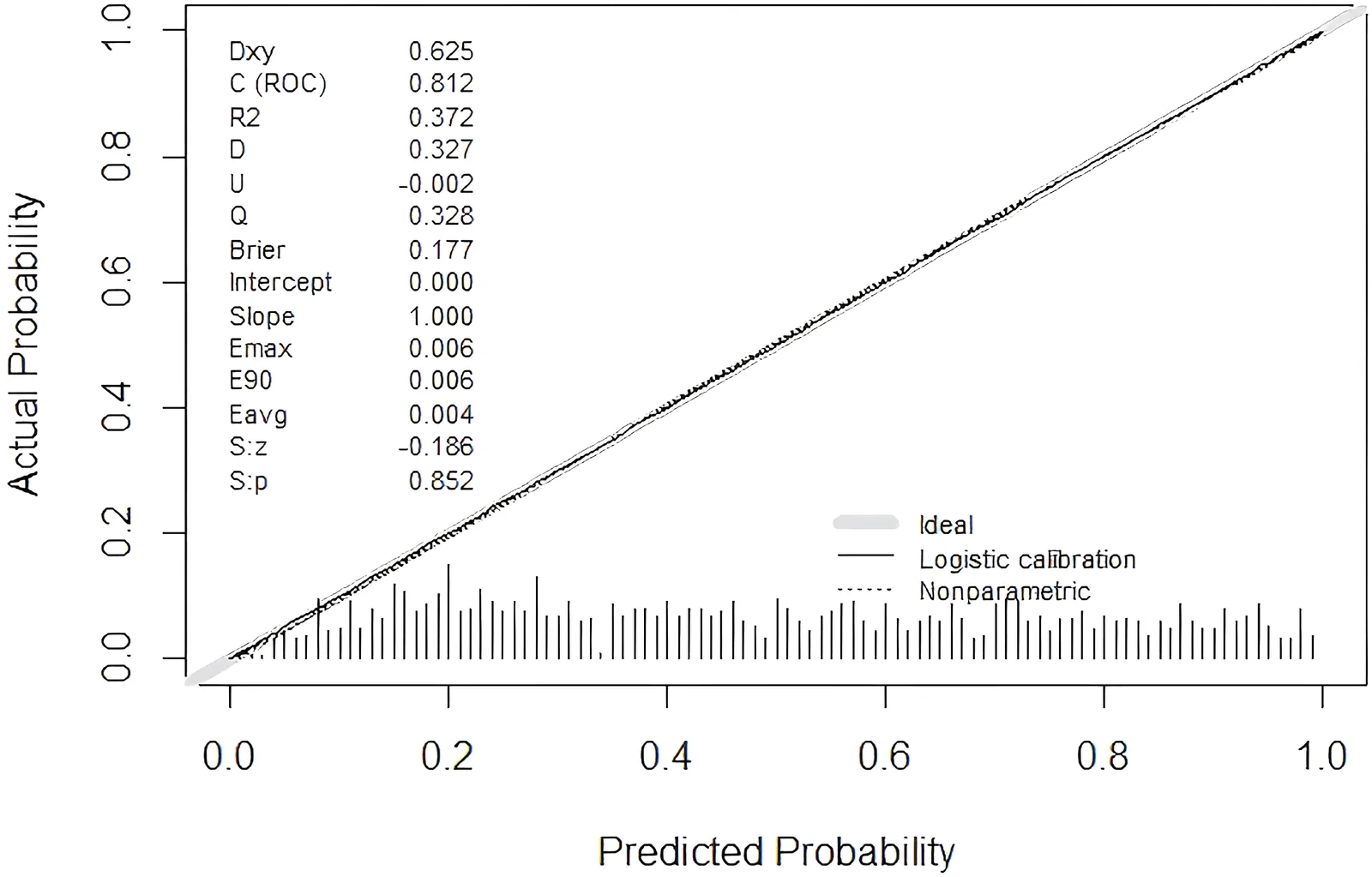
The calibration curve illustrates strong agreement between model-predicted probabilities and actual probabilities, with a Brier score of 0.177 indicating low overall prediction error. The curve closely approximates the ideal diagonal line (Slope = 1.000, Emax = 0.006), and no significant deviation was observed in the Hosmer-Lemeshow test (p = 0.852). The model demonstrates superior discrimination (C-index = 0.812) and a Dxy value of 0.625, further supporting its clinical utility.

This study validated the predictive accuracy of the model through calibration curves (**Figure 4**), which revealed a high degree of concordance between predicted probabilities and observed outcomes (Brier score = 0.177, calibration slope = 1.000) with no significant systematic deviation (Hosmer-Lemeshow test, p = 0.852). Coupled with excellent discriminative ability (C-index = 0.812), the model exhibited robust performance in risk stratification. Future work should further optimize calibration characteristics through external cohort validation and explore dynamic adjustment mechanisms to enhance clinical applicability.

**Figure 5:**
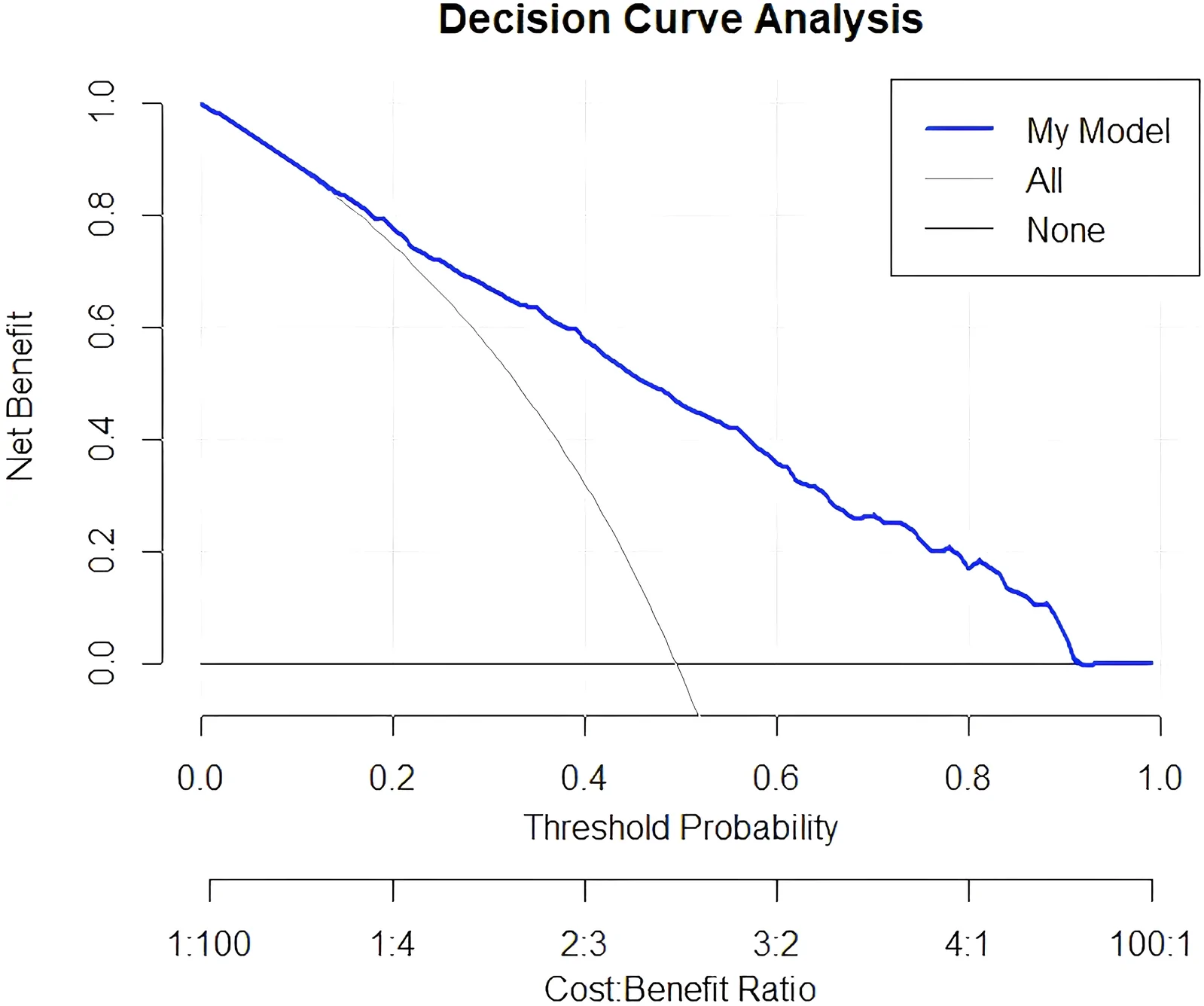
The DCA curve demonstrates acceptable model performance within the threshold probability range of 0.3–0.8, where it lies above both the “None” and “All” reference lines. The “All” line, representing the scenario where all samples are considered positive and receive intervention, displays a net benefit curve with a negative slope. The “None” line, indicating no intervention for any sample, yields a net benefit of zero.

The risk prediction model developed in this study underwent validation for clinical utility through decision curve analysis (DCA, **Figure 5**). Within the threshold probability range of 0.3 to 0.8, the model consistently demonstrated substantial positive net benefits and exhibited a clear distinction from the “ALL” and “NONE” reference lines. This suggests its capability to effectively balance false-positive and false-negative risks, thereby enhancing clinical decision-making. Additionally, with an impressive discrimination (C-index = 0.812) and calibration performance (Brier score = 0.177), this model serves as a dependable tool for personalized risk assessment. Future investigations should focus on validating its applicability in external cohorts and examining dynamic threshold adjustment mechanisms to tailor the model to various clinical contexts.

Subgroup analyses revealed population heterogeneity in the effects of both Ca/Mg ratio and Ca×IP product (**Table 3**). Gender stratification demonstrated stronger associations in females for Ca/Mg (OR = 10.012 vs. males: 6.983; both *P* < 0.001) and Ca×IP (OR = 2.570 vs. 2.009). Renal function stratification identified the strongest risk associations in the eGFR >90 mL/min/1.73 m² subgroup: Ca/Mg reached OR = 10.452 (95% CI: 5.402–20.223), significantly higher than in the 60–90 mL/min/1.73 m² group (OR = 4.599; P = 0.001). Of note, the subgroup with eGFR 30–60 mL/min/1.73 m² comprised individuals without a formal diagnosis of chronic kidney disease (since such patients were excluded from enrollment). In this small and heterogeneous subgroup, the association was non-significant for Ca/Mg (P = 0.072) and Ca×IP (OR = 2.363, P = 0.293). This dose-response attenuation suggests that calcium metabolism abnormalities predominantly influence stone formation during the renal compensatory phase (eGFR >60 mL/min/1.73 m²).

**Table 3:** Subgroup analyses of the association between serum Ca/Mg ratio, Ca×IP product, and nephrolithiasis risk, stratified by gender and eGFR.

Ca/Mg	Crude OR	95% CI	P-value
Gender			
Male	6.983	3.735-13.054	<0.001
Female	10.012	4.385-22.859	<0.001
eGFR, mL/min			
30~60	10.185	0.814-127.375	0.072
60~90	4.599	1.823-11.602	0.001
>90	10.452	5.402-20.223	<0.001
Ca×IP	Crude OR	95% CI	P-value
Gender			
Male	2.009	1.471-2.743	<0.001
Female	2.570	1.615-4.090	<0.001
eGFR, mL/min			
30~60	2.363	0.476-11.740	0.293
60~90	1.861	1.110-3.120	0.018
>90	2.208	1.607-3.034	<0.001

## Discussion

This case-control study investigates the association between serum electrolyte imbalance and nephrolithiasis. The multifactorial predictive model suggests that Ca/Mg, Ca×IP, and serum potassium are core interrelated electrolyte markers associated with stone formation, with risk associations substantially exceeding those of traditional isolated indicators (e.g., serum calcium/phosphate). These findings provide critical insights for identifying “metabolically imbalanced but biochemically normal” individuals in clinical practice. Crucially, a contribution of this study is the use of routine peripheral serum biomarkers to construct a composite electrolyte-based risk model. This serves as a highly accessible and practical alternative early-warning system in clinical scenarios where obtaining 24-hour urine samples is challenging or unfeasible.

The multivariable-adjusted OR for Ca/Mg reached 5.501 (95% CI: 3.105–9.745), exceeding the modest single-factor association of serum calcium (OR = 2.453, P = 0.187). This highlights the superior pathological relevance of Ca/Mg equilibrium over absolute ion concentrations. Supporting these results, molecular dynamics simulations conducted by Julie M. Riley’s research team demonstrate that magnesium can destabilize calcium oxalate ion pairs and reduce the size of calcium phosphate aggregates in a concentration-dependent manner (Massey, 2005; Khan et al., 2016). Interestingly, higher serum magnesium was inversely associated with nephrolithiasis in univariate analysis (OR = 0.001), although serum magnesium did not remain an independent predictor in the final multivariable model. This suggests that magnesium deficiency may primarily affect calcium-magnesium homeostasis rather than acting as an independent risk factor in our dataset. Consequently, there is a pressing need to reconsider magnesium supplementation approaches. Strategies that integrate monitoring of calcium metabolism alongside targeted interventions in TRPM6/7-mediated Ca/Mg cotransport could enhance therapeutic effectiveness (Schlingmann and Gudermann, 2005; Schlingmann et al., 2007; Tavasoli et al., 2019; Li et al., 2024; Vittori et al., 2024).

Despite comparable serum phosphate levels between groups (1.14 vs. 1.11 mmol/L, P = 0.103), elevated Ca×IP in the stone group (2.42 vs. 2.26 mmol²/L²) demonstrated independent risk significance (aOR=1.822). This implies that even with normal-range phosphate, calcium levels >2.35 mmol/L may drive Ca×IP beyond crystallization thresholds. Compensatory phosphate reduction under hyperparathyroidism or vitamin D hyperactivity likely maintains elevated Ca×IP through calcium-dominated effects, underscoring the clinical value of routine Ca×IP monitoring, particularly for individuals with high-normal calcium (Ketha et al., 2015; Taylor et al., 2015; Islam et al., 2020; Zhu et al., 2023).

While our serum-only data cannot directly establish these mechanisms, the following pathways have been proposed in the literature and may help explain the observed associations: Hypokalemia (aOR=0.128) may be associated with Ca/Mg/P dysregulation through several potential mechanisms, including: (1) Metabolic acidosis induction via osteoclast RANKL/OPG signaling activation, increasing bone calcium release (Khalaf and Almudhi, 2022; Liu et al., 2024); (2) Renal calcium handling disruption, elevating urinary calcium excretion (Caudarella and Vescini, 2009; Magni et al., 2021; Ferraro et al., 2024); (3) Mg²^+^-K^+^ vicious cycle: magnesium deficiency impairs Na^+^/K^+^-ATPase, exacerbating renal potassium wasting (Aronsen et al., 2015; Pirkmajer and Chibalin, 2019; Walker, 2019). Again, these mechanistic interpretations are speculative and require validation in experimental or urinary biomarker studies. This “hypomagnesemia-hypokalemia” synergy reduces calcium-sensing receptor sensitivity, triggering uncontrolled PTH secretion and establishing a self-reinforcing “hypercalcemia-hypercalciuria” loop, which may help explain the relatively strong association of hypokalemia in our multivariable model.

The model integrates metabolic balance indices (Ca/Mg, Ca×IP, K^+^) with traditional parameters (BMI, Cl^-^, Cr, Hb), which may help identify “covert imbalance” in calcium metabolism through compensatory mechanisms. Despite comparable serum calcium between groups (2.23 vs. 2.23 mmol/L, P = 0.996), Ca/Mg and Ca×IP sensitively detected subclinical dysregulation—minor Mg/IP fluctuations amplified calcium’s pathological impact. These composite biomarkers overcome the limitations of single-ion testing, serving as early clinical indicators of metabolic decompensation.

Persistent BMI significance (overweight aOR=1.585) may reflect adipose-endocrine disruption. Specifically, leptin upregulates renal NHE3 (promoting acid load) while suppressing vitamin D activation via PPARγ. This interplay may establish an “obesity-acidosis-mineral disorder” axis (Lovegrove et al., 2023). Hyperchloremia (aOR=1.064) and anemia (Hb aOR=0.985) further suggest acid-base imbalance and chronic inflammation indirectly modulate crystallization (Kadowaki et al., 2022).

Although elevated creatinine retained significance (aOR=1.048), its effect attenuated with declining eGFR (eGFR>90: OR = 10.452 vs. eGFR 30–60: P = 0.072). This “compensatory-phase predominance” can be attributed to contrasting mechanisms at work. In the early stages of CKD, the kidneys retain their ability to concentrate tubular fluids, leading to a higher risk of calcium phosphate supersaturation. In contrast, during the later stages of CKD, the development of secondary hyperparathyroidism obscures the influence of primary metabolic factors (Lindberg, 2005; Islam et al., 2020). This observation highlights the critical importance of this composite electrolyte model, particularly for early interventions aimed at leveraging the renal compensatory phase.

The model achieved an AUC of 0.812 (95% CI: 0.789–0.836). The model’s discriminative effectiveness likely reflects biological integration, as Ca/Mg and Ca×IP capture cross-pathway interactions, combined with the ability of the logistic model to weigh these composite indices along a log-odds scale. In this study, the nomogram we developed combines essential indicators from this electrolyte panel (Ca/Mg, Ca×IP) with traditional metrics like BMI and creatinine levels, allowing for a dynamic visual assessment of nephrolithiasis risk (C-index=0.812). One of the features of this nomogram is its scoring system for potassium and hemoglobin, which reflects the relative contribution of these variables in the final model, underscoring the potential clinical importance of a proposed metabolic interplay involving hypokalemia, anemia, and acid-base status, though this interaction is not directly demonstrated by our data. This provides a practical framework for applying this multi-indicator approach in clinical settings. Calibration curves and decision curve analysis further validated the model’s reliability and revealed its clinical net benefit. With this tool, clinicians may be better supported in risk stratification and targeted monitoring of high-risk individuals (score ≥240) by utilizing interventions informed by these electrolyte-based insights.

We acknowledge that 24-hour urine metabolic evaluation remains the gold standard for stone risk assessment. Our serum-based indices reflect systemic electrolyte balance rather than direct urinary supersaturation, and we did not measure urinary electrolytes in this cohort. Therefore, the nomogram should be viewed as a preliminary screening tool, not a replacement for urine testing. We also did not compare the performance of our nomogram directly with 24-hour urine profiles or other existing risk tools. The added clinical value of this serum-based model relative to or in combination with urine testing remains unknown and should be evaluated in future prospective studies correlating serum and urinary indices.

A key contribution of this model is its potential to capture subclinical metabolic disturbances. For example, it can classify individuals with normal serum calcium but abnormal Ca/Mg and Ca×IP as being at elevated risk. This capacity arises from synergistic interactions within this electrolyte panel. This “individual-normalized but system-dysregulated” phenomenon, if confirmed in prospective studies, might help explain recurrent stone formation in patients with unremarkable conventional test results, an area where traditional single-marker systems offer limited insight.

## Limitations

This study has several limitations. First, its retrospective, case-control design precludes the establishment of causality. Our findings can only describe associations, not causal relationships. The observed serological alterations might partly be attributed to secondary changes in patients’ dietary habits following the onset of stone disease, or to the disease process itself. Future prospective cohort studies are necessary to clarify the temporal and causal direction of these associations. Second, we did not collect information on dietary habits (including water and calcium intake), use of diuretics, vitamin D or calcium supplements, or medications affecting bone metabolism. These unmeasured factors could confound the associations observed. Future prospective studies with detailed dietary and medication records are needed to address this limitation. Our subgroup analysis included individuals with eGFR 30–60 mL/min/1.73 m² who did not have a formal CKD diagnosis; however, the small sample size and heterogeneity in this subgroup limit interpretability. Third, the diagnostic accuracy of this metabolic approach falls short when compared to imaging techniques, particularly urinary tract ultrasonography, which is widely regarded as the gold standard for detecting nephrolithiasis. Moreover, the retrospective nature of this study introduces potential selection bias, which could skew our findings. Fourth, the absence of 24-hour urinary biochemical data limited in-depth analysis of serum-urine metabolic correlations. Fifth, and perhaps most importantly, this study lacks data on stone composition analysis. Our current model primarily captures a generalized dysregulation of electrolyte homeostasis. Given that nephrolithiasis encompasses highly heterogeneous pathologies, future studies must incorporate subgroup validation specifically tailored to distinct stone compositions (e.g., calcium oxalate versus uric acid) to provide more precise and individualized nutritional guidance.

## Data Availability

The original contributions presented in the study are included in the article/supplementary material. Further inquiries can be directed to the corresponding author.
